# Size-Dependent Antibacterial Activity of Silver Nanoparticle-Loaded Graphene Oxide Nanosheets

**DOI:** 10.3390/nano10061207

**Published:** 2020-06-20

**Authors:** Thi Tuong Vi Truong, Selvaraj Rajesh Kumar, Yu-Tzu Huang, Dave W. Chen, Yu-Kuo Liu, Shingjiang Jessie Lue

**Affiliations:** 1 Department of Chemical and Materials Engineering, Chang Gung University, Taoyuan City 33302, Taiwan; truongthituongvi005@gmail.com (T.T.V.T.); rajeshkumarnst@gmail.com (S.R.K.); ykliu@mail.cgu.edu.tw (Y.-K.L.); 2 Department of Chemical Engineering, Chung Yuan Christian University, Taoyuan City 32023, Taiwan; yt_huang@cycu.edu.tw; 3 Department of Orthopedic Surgery, Chang Gung Memorial Hospital, Keelung City 20445, Taiwan; mr5181@cgmh.org.tw; 4 Department of Safety, Health and Environment Engineering, Ming-Chi University of Technology, New Taipei City 24301, Taiwan

**Keywords:** graphene oxide, silver nanoparticles, size effect, antibacterial activity, reactive oxygen species

## Abstract

A series of graphene oxide (GO) suspensions with different particle sizes (<100 nm, ~100 nm, ~1 µm and >1 µm) were successfully fabricated after 0, 30, 60 and 120 min of sonication, respectively. The antibacterial properties of GO suspensions showed that >1 µm GO size resulted in a loss of nearly 50% of bacterial viability, which was higher than treatment by ~100 nm GO size (25%) towards *Escherichia coli* (*E. coli*). Complete entrapment of bacteria by the larger GO was observed in transmission electron microscopy (TEM). Silver nanoparticles (Ag NPs) were doped onto GO samples with different lateral sizes to form GO–Ag NP composites. Resulting larger GO–Ag NPs showed higher antibacterial activity than smaller GO–Ag NPs. As observed by Fourier transform infrared spectroscopy (FTIR), the interaction between *E. coli* and GO occurred mainly at the outer membrane, where membrane amino acids interact with hydroxyl and epoxy groups. The reactive oxygen species (ROS) and the considerable penetration of released Ag^+^ into the inner bacterial cell membrane result in loss of membrane integrity and damaged morphology. The present work improves the combined action of GO size effect with constant Ag loadings for potential antibacterial activity.

## 1. Introduction

To date, the rapid development of bacterial infections has threatened health and life. However, antibiotic resistance is making some infections extremely difficult to treat [1]. This issue has resulted in an urgent demand for advanced nanomaterials [2,3]. To overcome this problem, several replacement materials for antibacterial treatment such as carbonaceous nanomaterials [4], metal nanoparticles and metal oxide nanoparticles [5] have been explored.

Graphene oxide (GO) is composed of single-atom-thick sheets of sp^2^-bonded carbon with oxygen-containing functional groups. It is a typical two-dimensional material made of carbon atoms in a honeycomb crystal lattice [6] and can be modified into various derivatives and composites [7]. GO is considered to be a “nanoknife” whose sharp edge can “cut” bacterial membrane to cause cell damage [8]. Mangadlao et al. reported on how the sharp edges of processed GO sheets can immobilize bacteria onto the GO sheet surface, preventing it from puncturing and developing [9]. Zhou et al. proved that the wrinkled surface of GO sheets contributed to the bactericidal effect [10]. In addition, GO exhibited antibacterial activity via chemical factors. For instance, Liu et al. proposed that GO can cause stress to bacterial membranes by oxidizing the cellular membrane structure [11]. Moreover, Kurantowicz et al. showed that GO can cause mechanical stress by direct attachment to the surface of bacteria [12]. Thereby, the functional groups of GO, including carboxyl groups (–COOH), epoxy groups (–C–O–C) which possibly bonded to, and consumed, bacteria [13].

The mechanism of GO antibacterial activity is still being explored. One noticeable issue with using GO in biological research is the particle size factor. Liu et al. reported that in the suspension phase, larger GO sheets (0.75 µm^2^) completely covered *E. coli*, resulting in much greater loss of bacterial viability than smaller GO sheets (0.01 µm^2^) [14]. Zhao et al. showed that larger GO sheets (0.5 to 3 µm) caused higher antibacterial activity. They showed scanning emission microscopy (SEM) images of *E. coli* membrane rupture and dispersion of the cytoplasm into the extracellular surroundings after exposure to large GO sheets [15]. Perreault et al. proved that large GO sheets exhibited higher antibacterial activity due to bacterial entrapment and inhibition of cell proliferation. Faria et al. prepared small-size debris GO, which showed a lower antibacterial effect than that of original-size GO. They proved that the loss of glutathione was lower when GO was fragmented into debris phase [16]. On the other hand, Jia et al. proved that small GO sheets exhibited higher antimicrobial activity towards *Tox2* bacteria than large GO sheets due to the induction of cytotoxicity via internalization [17].

In the GO-coated membrane phase, small GO sheets approximately 0.01 µm^2^ in size exhibited a more effective antimicrobial effect on *E. coli* than large GO sheets approximately 0.65 µm^2^ in size. One possible reason is that small GO sheets possess a higher defect density, which facilitates interactions between GO functional groups and bacterial membranes, leading to more bacterial death [18]. In addition, Dallavalle et al. hypothesized that the small GO sheets can pierce the membrane in a suitable geometric orientation, which was reproduced by computational studies [19]. In light of the inconsistent results of the GO size-dependent bactericidal effect, further study to investigate the size-dependent properties is needed. 

Modification of GO with nanomaterials enhances its antibacterial performance [20,21,22]. In particular, silver nanoparticles (Ag NPs) are among the effective novel metals in biomedical applications. Silver nanoparticles are well-known materials for good antibacterial activity behavior even at small concentrations [23]. Previous studies have demonstrated that the GO–Ag NP antibacterial activity benefits from the synergetic effects between GO and Ag NPs [24,25]. In the GO–Ag NPs, GO wraps the bacterial surface while Ag NPs penetrate into the inner cell or accumulate at the bacterial membrane, destroying the bacterial cells. The GO–Ag NPs synergistic effect was examined using a wide range of bacteria including gram-negative and gram-positive bacteria such as *Staphylococcus aureus* [24], *Pseudomonas aeruginosa* [26], *Bacillus subtilis* [27], *Klebsiella pneumonia* [28], etc. However, to the best of our knowledge, no research data regarding the antibacterial activity of GO sheets with different lateral sizes in GO–Ag NP composites have been reported yet.

To influence the GO size effects, we incorporate the constant loading of Ag NPs into different-sized GO nanosheets. Transmission electron microscope (TEM), dynamic light scattering (DLS) and inductively coupled plasma–optical emission spectrometry (ICP-OES) were the main techniques used to characterize the GO and GO–Ag NPs. Subsequently, time-dependent antibacterial activity and live/dead cell assays were used to compare the size-dependent bactericidal activity. Reactive oxygen species (ROS) assays, TEM and Fourier transform infrared spectroscopy (FTIR) of GO- and GO–Ag NP-treated *E. coli* were applied to determine the reasons for the above findings. Finally, we propose insight into the bactericidal mechanisms on different GO sizes for GO and GO–Ag NP functionality. 

## 2. Materials and Methods 

### 2.1. Materials

Graphite powder (G), potassium permanganate (KMnO_4_), hydrogen peroxide (H_2_O_2_), potassium bromide (KBr), hoechst 333242 (HS), glutaraldehyde 50%, sodium borohydride (NaBH_4_), 1% osmic acid, 2’,7′-dichlorodihydrofluorescein diacetate (DCFH-DA), propidium iodide (PI), dimethyl sulfoxide (DMSO) and silver nitrate (AgNO_3_) were purchased from Sigma-Aldrich, St. Louis, MO, USA. Sulfuric acid (H_2_SO_4_, 98%) was purchased from Lab Alley, Austin, Texas, USA. Hydrochloric acid (HCl) and nitric acid (HNO_3_) were purchased from Showa Chemical Industry Co., Ltd., Honshu, Japan. Luria-Bertani (LB) broth, phosphate-buffered saline (PBS) and agar-agar powder were purchased from Sigma-Aldrich, St. Louis, MO, USA. *E. coli* (DH5α) was purchased from Thermo Fisher Scientific Inc., Taipei City, Taiwan.

### 2.2. Synthesis of GO 

GO was fabricated according to the modified Hummer’s method with trivial modification [29]. Briefly, 3 g of graphite powder was dispersed in 400 mL of H_2_SO_4_ solution with continuous stirring for 10 min. Then, 3 g of KMnO_4_ was added. A total of five portions of KMnO_4_ were sequentially added once the solution changed from green to black. Subsequently, 500 g of deionized (DI) water was added to avoid increasing the temperature. The supernatant solution was discarded, and the residual gel-like products were washed with HCl and DI water using Benchtop centrifugation (Hermel Z236K, Sanchong, New Taipei City, Taiwan) under 10,000 rpm until a neutral pH was obtained. The final products were dried overnight in a vacuum oven at 60 °C. 

### 2.3. Synthesis of GO with Different Lateral Sizes 

Four batches of GO suspensions were prepared for various GO sizes. Each batch contained 1 mg mL^−1^ GO powder. The GO suspensions were sonicated for different times: 0, 30, 60 and 120 min using a probe sonicator Qsonica (Sunway Scientific Corporation, Hsinchu, Taiwan). An ice bath was used during the sonication process. The final GO products were kept in dry, cool areas for further analysis.

### 2.4. Synthesis of GO–Ag NPs with Different Latteral Sizes of GO

In short, 5 mL of GO solution at a concentration of 1 mg mL^−1^ was mixed with 15 mL of 1× 10^−3^ M AgNO_3_ for 30 min. Subsequently, 5 mL of a 3.35 × 10^−3^ M NaBH_4_ aqueous solution was added dropwise and continuously stirred for 1 h in an ice bath. The color of the solution slowly turned dark yellow, indicating the reduction in Ag^+^ ions and the formation of Ag NPs. The synthesized GO–Ag NP composites were washed many times with DI water via centrifugation at 10,000 rpm for 10 min. Final products were dialyzed using dialysis 2000 Da to remove unreacted Ag solution and/or Ag particles for 1 day before further analysis.

### 2.5. Characterizations

Two-dimensional GO and GO–Ag NP images were obtained using TEM (JEM 2000EXII, JEOL, Tokyo, Japan). Sample structures were analyzed using X-ray diffraction (XRD, Siemens D5005, Oslo, Norway) to determine the element structure and phase purity. The 2θ diffractogram was scanned in the range of 5–80° at a scan rate of 4° min^−1^ with Cu-Kα radiation. FTIR (Horiba FT-730G, Kyoto, Japan) was used to identify the functional groups of the sample in the range of 500–4000 cm^−1^. A UV-visible spectrophotometer (V-650, Tokyo, Japan) was used to measure the change in the lateral size of GO and GO–Ag NP with various absorption wavelengths. The zeta potential and size distribution of GO and GO–Ag NPs were recorded in triplicate via a light scattering instrument (Zetasizer, 2000 HAS, Malvern, Worcestershire, UK). The Ag content was analyzed using inductively coupled plasma optical emission spectrometry (ICP-OES, Agilent 700 Series ICP Optical Emission Spectrometers, Victoria, Australia).

### 2.6. Antibacterial Test

#### 2.6.1. Time-Dependent Antibacterial Activity Assay

Optimal readings were taken to examine the bacterial growth when GO or GO–Ag NPs was presented. The GO suspensions were stable up to 13 h. Before the antibacterial experiments, *E. coli* colonies were cultured overnight in LB broth at 37 °C using an orbital shaker incubator (Firstek S300R Datong, Taipei City, Taiwan) and gently centrifuged at 2000 rpm for 5 min using a benchtop centrifuge. The supernatant was removed. The precipitate was collected and then diluted with LB broth to achieve an optical density of 0.3. The above bacterial suspensions were then transferred into 48-well plates. Approximately 50 µL of each sample suspension was added to 450 µL of LB broth, and the plates were shaken for 1 h. Pristine LB broth without treatment was used as a control sample. Ten microliters of the sample mixture was removed and transferred into 96-well plates. Subsequently, 90 µL LB broth was added to each well. Triplicate optical density readings were measured using a microplate reader (Biotek, Hong Kong, China) at a wavelength of 600 nm. Data were read at intervals until 13 h for GO and 4 h for GO–Ag NP samples. The inhibition percentage was calculated via the following equation:

$$\mathrm{Inhibition}\;(\%)=\left(1-\frac{OD\;value\;of\;sample\;at\;time\;t\;–\;OD\;value\;of\;sample\;at\;time\;0}{OD\;value\;of\;control\;at\;time\;t-OD\;value\;of\;control\;at\;time\;0}\right)\times 100$$

#### 2.6.2. Bacterial Morphology of GO and GO–Ag NP Treatments

In short, a 100 mg µL^−1^ of 10^6^ CFU mL^−1^ bacterial suspension was incubated with the smallest and largest GO sheets for 1 h. The mixture was fixed with 4% glutaraldehyde for 1 h and washed twice with PBS. The bacterial suspension was postfixed with 1% osmic acid for 45 min and washed twice with PBS. The samples were dehydrated with gradient ethanol (20%, 40%, 60%, 80%). The samples were then dropped on a gold grid for TEM observation. 

#### 2.6.3. Live/Dead Cell Assay

The *E. coli* suspensions were diluted with PBS solution (to achieve an OD value close to 0.1) before treatment with the smallest and largest GO sheets (100 μg mL^−1^) for 2 h of incubation. A mixture containing 5 µL of HS and 1 µL of PI was added to the bacterial suspension. These mixtures were then incubated for 15 min in the dark and washed twice with PBS solution. A confocal laser scanning microscope (CLSM, Zeiss LSM 510-Meta, Heidelberg, Germany) was used to observe the live/dead cells.

#### 2.6.4. Reactive Oxygen Species 

Theoretically, the intracellular ROS was measured based on the intracellular peroxide-dependent oxidation of 2′,7′-dichlorodihydrofluorescein diacetate (DCFH-DA) to form the fluorescent compound 2′,7′-dichlorofluorescein (DCF) [30]. Once materials have high ROS production, the green color was detected. Therefore, DCFH-DA assay was used in this study. A DCFH-DA stock solution was prepared in DMSO solution. *E. coli* suspensions with an OD close to 0.1 were treated with 100 μg mL^−1^ control, GO and GO–Ag NP solutions with the smallest and largest lateral sizes for 2 h of incubation. The mixture was added to 100 µL of 25 µM DCFH-DA without shaking. Those mixtures were kept in the dark for 30 min at 37 °C. The samples were washed twice using PBS. Fluorescence images were obtained using a CLSM at an emission of 535 nm and excitation of 485 nm. The ROS level was evaluated by green color production.

## 3. Results

### 3.1. Characterization of GO with Different Sizes

**Figure 1:**
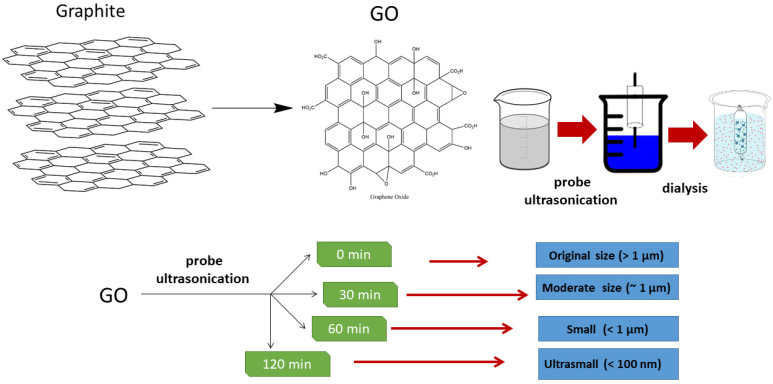
Preparation of GO from graphite and manufacturing of different GO sizes via probe sonication.

**Figure 2:**
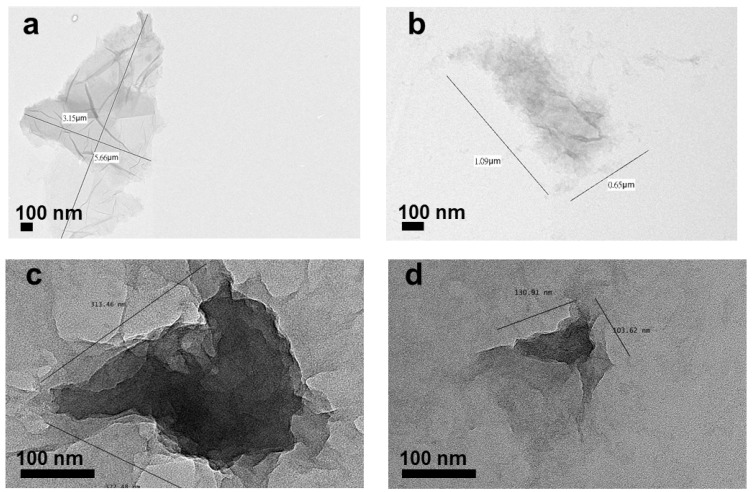
TEM images of GO samples with various sizes: (**a**) >1 µm, (**b**) ~1 µm, (**c**) 100 nm to 1 µm and (**d**) ~100 nm.

The preparation process is illustrated in Figure 1. After sonication, the size of GO can be categorized as very large (original size) (>1 µm), moderate (~1 µm), small (<1 µm) and ultrasmall (~100 nm). The size was determined by a DLS analyzer (Table 1). The size variation of GO was clearly observed under TEM (Figure 2a–d). The size determined by DLS might be larger than that calculated by TEM. This is probably attributed to the fact that GO has a non-spherical shape and extends in various directions. The DLS measurement assumes the particles are spherical in shape. This instrument may not be able to detect small particles below the detection limit. In theory, the light scattered by a particle depends upon the shape, size and molecular weight, as well as the refractive indices, of the particle. The scattered light from individual particles is interrupted by the scattered light from other particles before reaching the detector, since the molecules are in random movement due to Brownian motion, which will result in random fluctuations in time [31]. DLS data usually exhibited larger values than TEM due to the movement of the nanoparticles when they were dispersed in solution [32]. Although the DLS and TEM analyses showed different size values, the data were positively correlated.

**Figure 3:**
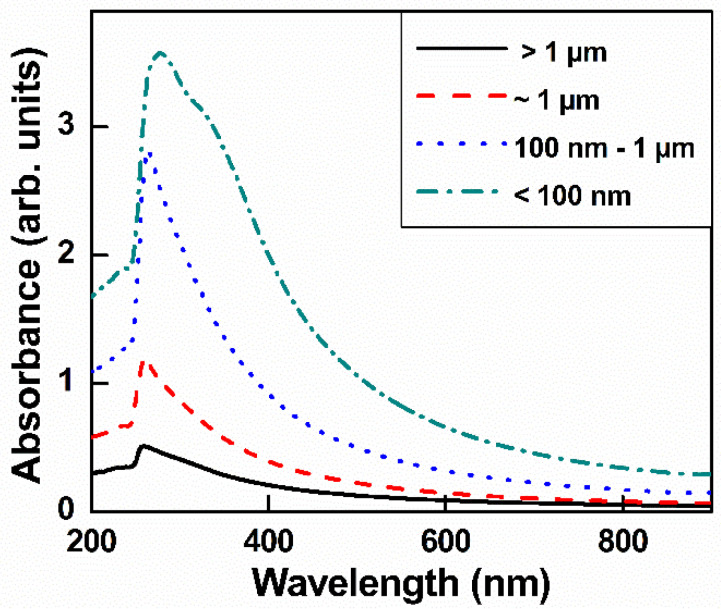
UV-visible spectra of GO with different sizes in aqueous solution.

Figure 3 shows the UV-visible spectrum of GO with different lateral sizes. The GO spectrum has a typical shoulder and main peak: a shoulder peak attributed to π–π * bonding and attributed to *n–π* * bonding. When GO was subjected to ultrasonication, the additional oxygen between GO layers made it easier to exfoliate; therefore, the smaller GO had a higher intensity value than the larger GO. This result was consistent with a previous report [33].

As shown in Figure S1a, the FTIR results have proven the characteristics of GO domain groups [29]. The GO spectrum showed characteristic peaks at 3404 (C–OH), 1733 (C=O), 1622 (the vibration of C=C domains), 1220 (C–O) and 1055 cm^−1^ (C–O stretching). Based on the presence of these oxygen-containing groups, it was confirmed that these groups resulted from the GO oxidation process. This FTIR result is similar to that of a previous study [34]. Moreover, the XRD pattern showed a typical peak at 11.7° (Figure S1b), indicating that GO was successfully prepared. The zeta potential of GO of various sizes did not fluctuate much (−30~−37 mV, Table 1), which indicated that GO remains moderately stable after the sonication process. The negative zeta potential values originated from the carboxylate functional groups in GO and were not size-dependent.

### 3.2. Characterizations of GO–Ag NPs Corresponding to Different Sizes of GO

**Figure 4:**
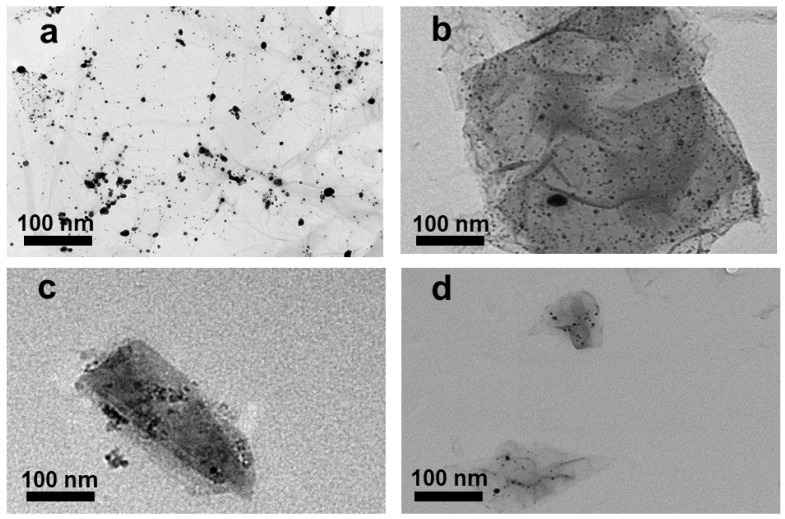
TEM images of GO–Ag NPs with various GO sizes (**a**) >1 µm, (**b**) ~1 µm, (**c**) 100 nm to 1 µm and (**d**) ~100 nm.

**Figure 5:**
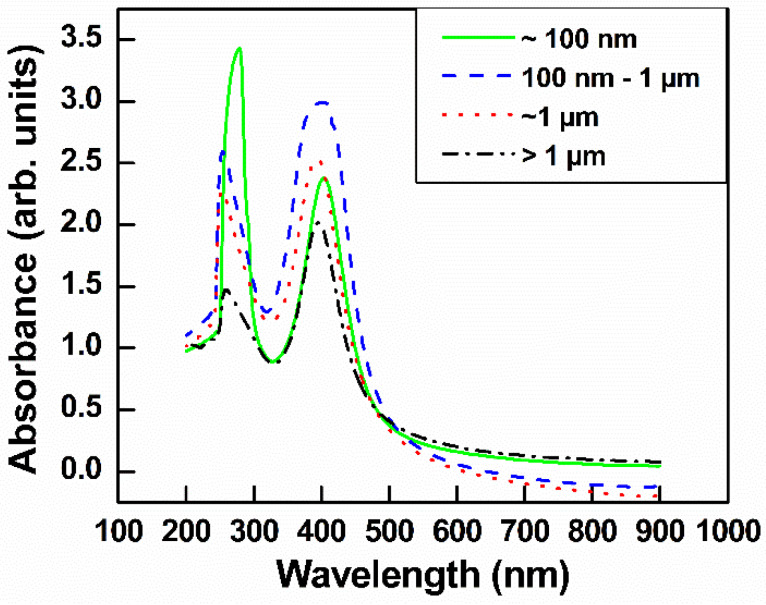
UV-visible spectra of GO–Ag NPs with various sizes GO.

GO–Ag NPs with different GO sizes were fabricated using NaBH_4_ as both a stabilizer and a reduction agent. TEM images of GO–Ag NPs with different sizes of GO are shown in Figure 4a–d. Obviously, the Ag NPs were uniformly anchored on the GO layers, regardless of the GO size. The UV spectra of the GO–Ag NP samples showed two main peaks, 230 nm attributed to GO and 400 nm attributed to Ag NPs [35] (Figure 5). Obviously, the peak intensity at 230 nm for the smaller GO–Ag NPs was higher than that for the larger GO–Ag NPs. The trend is similar to that in GO solutions (Figure 3).

The characterization of GO–Ag NPs using FTIR and XRD is shown in Figure S2. According to Figure S2a, FTIR analysis showed that the intensities of GO characteristic peaks significantly decreased. This result suggested that the hydroxyl groups in GO may react with Ag [36]. The grafting of Ag NPs onto GO sheets was also analyzed using XRD analysis. According to Figure S2b, the GO–Ag NP composite gave rise to the main XRD peaks at 38.1°, 44.3°, 64.5°, and 77.5°, corresponding to the (111), (200), (220), and (311) planes, respectively and confirming the face-centered cubic structure of Ag. However, the main peak of GO (2θ of 10°) disappeared in the spectra of the GO–Ag NP samples, probably due to the change in lattice position [37]. Hui et al. proved that the disappearance of GO peaks due to the incorporation of GO and Ag NPs worked as spacers, avoiding the restacking of GO layers [38,39]. Zhao et al. reported similar finding in layered graphite oxide preparation [40].

**Figure 6:**
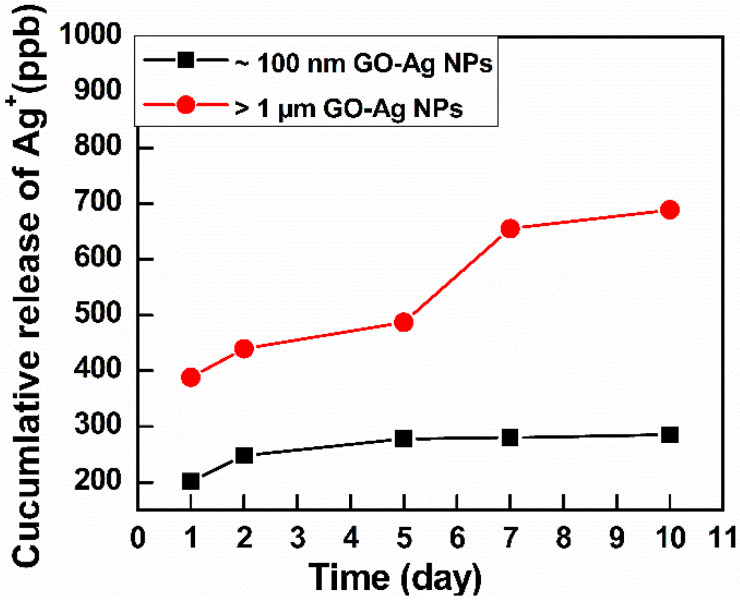
Ag^+^ release analysis by ICP-EOS of GO–Ag NPs with GO sizes >1 µm and ~100 nm.

As indicated in Figure 6, the initial Ag^+^ content in the largest GO–Ag NPs was approximately 400 ppb, higher than that in the smallest GO–Ag NPs (approximately 200 ppb). The Ag^+^ content increased over time. On the 5th day, the Ag^+^ content in small GO–Ag NPs reached 285 ppb and remained stable until the 10th day. On the other hand, Ag^+^ in the largest GO–Ag NPs was released rapidly, and a maximum content of 688 ppb Ag^+^ was achieved at day 10.

### 3.3. Antibacterial Activity of GO with Different Sizes 

**Figure 7:**
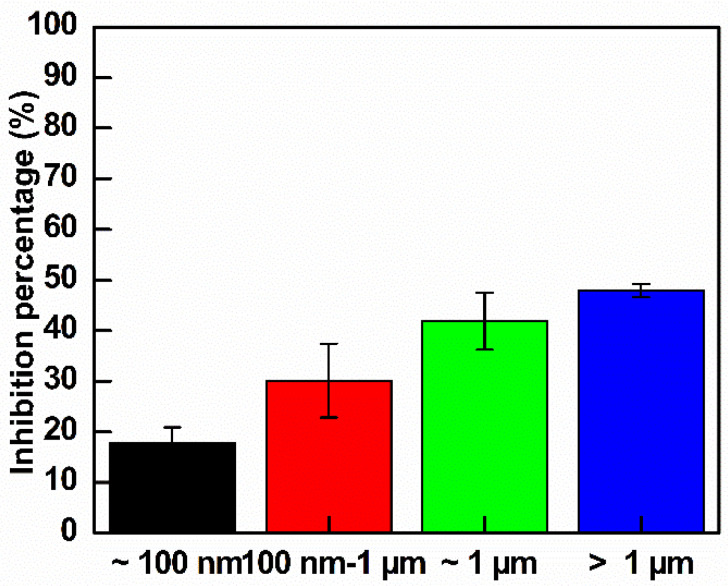
Inhibition rates of *E. coli* control and treated GO of different sizes after 13 h of observation (*n* = 3).

Initially, *E. coli* suspensions were cultured with LB broth until reaching log phase and were diluted to an OD value of approximately 0.15. Bacteria were treated with different GO sizes at the same concentration of 300 µg mL^−1^. The growth curves are shown in Figure S3a, and the calculated inhibition percentages are shown in Figure 7. According to Figure 7, the trend of inhibition percentage steadily increased from the smallest GO to the largest GO. The quantitative inhibition percentages after 13 h of treatment were 17.8%, 30.1%, 41.8% and 47.9% for ~100 nm, 100 nm to 1 µm, ~1 µm and >1 µm GO, respectively. There is a significant difference between >1 µm and 100 nm GO in the above inhibition percentage, whereas the inhibition between >1 and ~1 µm is similar. Note that the size of *E. coli* was from 0.5 to 2 µm, so that the larger GO size totally trapped the entire *E. coli* cell, producing higher damage to *E. coli*. 

**Figure 8:**
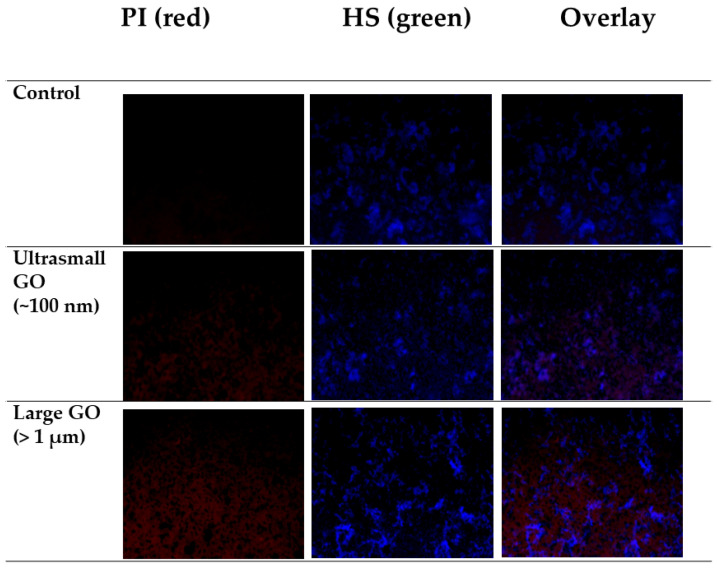
Live/dead cell staining by PI and HS of *E. coli* control culture and cultures subjected to treatments with >1 µm and ~100 nm GO.

To evaluate the number of live/dead cells, fluorescence images were captured by CLSM using PI as the dead cell indicator and HS as the live cell indicator. As shown in Figure 8, the majority of bacteria in the control groups were still alive. After being exposed to small GO, some bacteria were damage, as showed in red color intensity increase. In the larger GO sample, there was a significant reduction in the extent of blue color in the images, whereas the extent of red color was increased. These results also confirmed that the antibacterial activity of the largest GO was higher than that of the smallest GO. 

**Figure 9:**
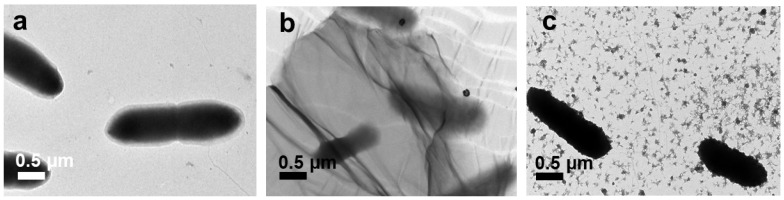
TEM images of the *E. coli* control cells (**a**) and cells treated with >1 µm (**b**) and ~100 nm (**c**) GO.

Under TEM analysis, the control *E. coli* appeared as typical smooth, rod-shape cells, as shown in Figure 9a. Obviously, *E. coli* cells were trapped by large GO (Figure 9b) and experienced serious damage. The >1 µm GO completely covered the *E. coli* cells, destroying the integrity of their morphology. This was because by entrapping the *E. coli* cells, the GO sheets isolated the *E. coli* colony from the nutrient medium and inhibited *E. coli* development. Liu et al. proved that the development of bacteria was restrained by a cell entrapment mechanism [11]. However, the ~100 nm GO sheets were merely attached to the surrounding bacterial membrane (Figure 9c) by van der Waals forces between the sp^2^ interlayers in GO and the lipid bilayer of the bacterial cell membranes without stressing the whole *E. coli* cells (Figure 9c) [41]; therefore, the *E. coli* morphology after treatment with ~100 nm GO sheets was not severely damaged. 

**Figure 10:**
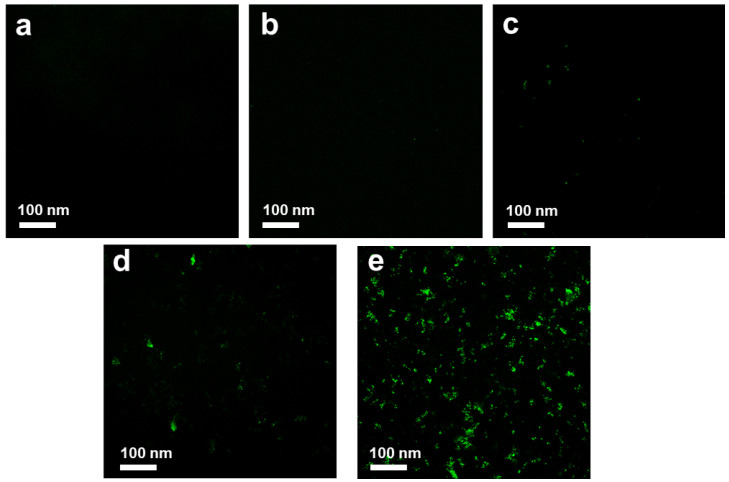
Reactive oxygen species (ROS) production of control (**a**), ~100 nm GO (**b**), >1 µm GO (**c**), ~100 nm GO–Ag NPs (**d**) and >1 µm GO–Ag NPs (**e**) at the same 2 h incubation time, the bacterial inhibition rates are 0.5%, 0.8%, 5.1% and 21.43% for samples (**b**)–(**e**), respectively.

DCFH-DA was used as an indicator for ROS detection. As shown in Figure 10a, the control sample was dark in color. GO with a size of ~100 nm also showed no ROS signal (Figure 10b), whereas >1 µm GO exhibited weak green fluorescence, as indicated in Figure 10c. The low ROS production indicated that GO does not have a high impact on oxidative stress in *E. coli*. This result was in agreement with the results of Liu et al., who proved that the production of ROS induced by >1 µm GO was significantly higher than that induced by ~100 nm GO [14]. In comparison, ~100 nm GO sheets showed weaker antibacterial activity because they only adhered to the bacterial surface without efficiently isolating bacteria from the growth medium. Faria et al. prepared small-size debris GO, which showed lower antibacterial effects than that of original-size GO. They proved that the loss of glutathione was lower when GO was fragmented into debris phase [16].

**Figure 11:**
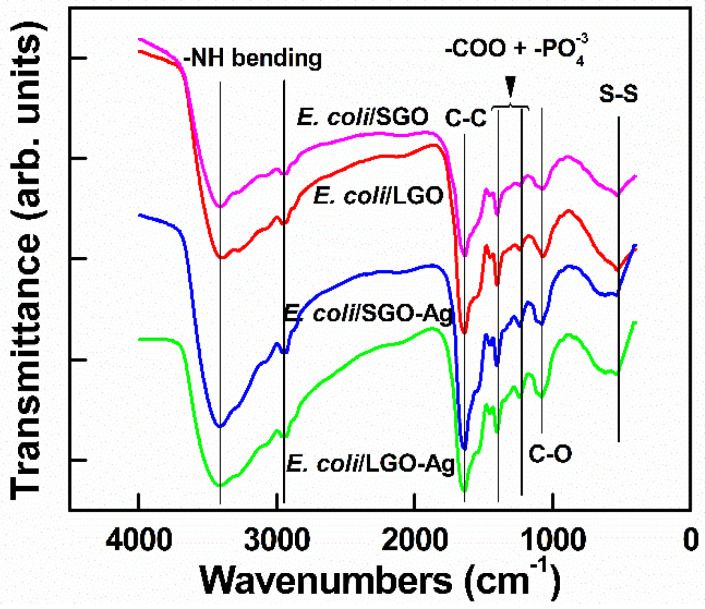
Fourier transform infrared spectroscopy (FTIR) characterizations of *E. coli* treated with ~100 nm GO, >1 µm GO, ~100 nm GO–Ag NPs and >1 µm GO–Ag NPs.

To investigate the bonding between bacteria after treatment with GO, FTIR spectroscopy was conducted. As shown in Figure 11, the peak at approximately 3413 cm^−1^ was ascribed to stretching of the –NH moiety of the amino acid after reacts with –OH in GO [42]. A significant peak at 1629 cm^−1^ was associated with secondary amides in the *E. coli* structure. On the other hand, the peaks at 1236 and 1409 cm^−1^ were attributed to the noncovalent bonding that occurred between –COOH and $-{\mathrm{PO}}_{4}^{-3\;}$ [43]. The peak at 520 cm^−1^ was attributed to disulfide bonding (S–S) adherence between GO and *E. coli* after treatment, indicating that ions were formed in the outer membrane [44]. Therefore, the interaction of >1 µm and ~100 nm GO with *E. coli* mainly occurred via GO’s functional groups, wherein –OH and –COOH react with amino acids in the bacterial membrane. This research was in agreement with that of Efremova et al., who reported that GO sheets and *E. coli* cells interacted by means of a neutralization reaction between the bacterial membrane and functional group on the GO surface [45]. Camesano et al. proved that the adhesion between *E. coli* and GO arises from the negatively charged bacterial outer membrane and the deprotonated carboxylic acid groups on GO [46]. This noncovalent bonding interaction prevented the bacteria from accessing the nutrition medium; therefore, the membrane was physically damaged [41]. On the other hand, when *E. coli* was incubated with GO under shaking, the *E. coli* was cleaved by biochemical reaction. This may possibly place *E. coli* under stress or physical damage. Therefore, the antibacterial activity of >1 µm GO was significantly higher than that of ~100 nm GO, and the proposed mechanism of GO antibacterial activity mainly involves physical wrapping, thus resulting in greater interaction with *E. coli*.

### 3.4. Antibacterial Activity of GO–Ag NPs with Different GO Sizes

**Figure 12:**
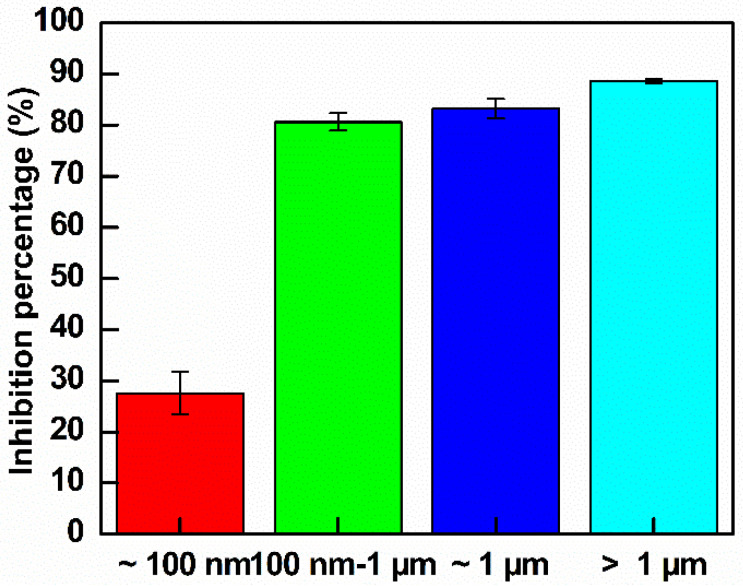
Inhibition rates of the control *E. coli* and treated *E. coli* with GO–Ag NPs with different GO sizes after 4 h of observation (*n* = 3).

*E. coli* suspensions were cultured in LB broth until reaching log phase and were then diluted to an OD value of approximately 0.15. The bacteria were treated with different sizes GO at the same concentration of 300 µg mL^−1^. The growth curve analysis was shown in Figure S3b and the calculated inhibition percentage was shown in Figure 12. As indicated in Figure 12, GO–Ag NPs larger than 100 nm exhibited good inhibition of *E. coli*. The quantitative inhibition percentages after 4 h of treatment were 27.6%, 80.6%, 83.2% and 88.6% for GO–Ag NPs ~ 100 nm, 100 nm–1 µm, ~1 µm and >1 µm, respectively. This study demonstrates a promising antibacterial effect of large GO-loaded Ag NPs. The largest GO–Ag NPs with a size of >1 µm had the greatest effect on the antibacterial activity.

Figure 10 shows that green fluorescence was clearly observed in *E. coli* treated with ~100 nm GO–Ag NPs, indicating that small Ag NP-loaded GO produced a moderate level of ROS (Figure 10d). Noticeably, as shown in Figure 10e, a green color was detected, which indicated that the ROS level produced by >1 µm GO–Ag NPs may be one of the reasons for the antibacterial activity.

Subsequently, the FTIR results in Figure 11 show that after treatment of *E. coli* with ~100 nm and >1 µm GO–Ag NPs, the spectral peaks were similar peaks to those of GO. However, the peaks at 520 cm^−1^ broadened and shifted to 620 cm^−1^, indicating that the metal interacted with the cell membrane [47].

**Figure 13:**
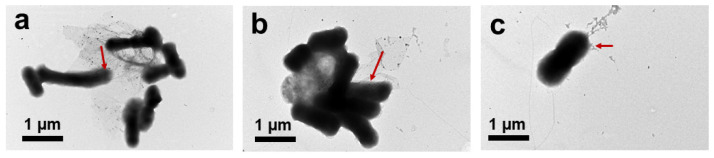
TEM images of E. coli after treatment with >1 µm (**a**), ~1 µm (**b**) and ~100 nm (**c**) GO–Ag NPs for 1 h incubation. Red arrow indicated for silver nanoparticles (Ag NPs) penetration into *E. coli*.

In summary, the greater antibacterial activity of GO–Ag NPs with large GO sheets than of GO–Ag NPs with small GO sheets can be explained in three perspectives. At first, the large GO could enhance the binding between GO and the *E. coli* membrane, resulting in more interaction upon *E. coli* treatment with large GO. The binding interactions and destruction of *E. coli* treatment with large GO–Ag NPs involve penetration of Ag into the bacterial membrane, as indicated by the TEM image in Figure 13. As shown in Figure 13, after treatment with different sized GO–Ag NPs, the *E. coli* morphology exhibited integrity structure damage. In particular, *E. coli* after treatment with >1 µm (Figure 13a) and ~1 µm GO–Ag NPs (Figure 13b) becomes damaged, clustered and gathered together. The single *E. coli* found after treatment with ~100 nm GO–Ag NPs (Figure 13c) did not show severe damage.

Another reason for the difference in antibacterial activity may relate to the amount of Ag^+^ released from small and large GO–Ag NPs. According to the ICP-OES results (Figure 6), the initial Ag content from the >1 µm GO–Ag NPs was higher than that from the ~100 nm GO–Ag NPs. Moreover, the release rate of Ag^+^ from the >1 µm GO–Ag NPs was significantly higher than that in ~100 nm GO–Ag NPs. Similar finding was also reported in the literature [48]. The release of nanoparticle ions generates an electrochemical potential and increases over time; these ions bind with thiol groups in the phospholipid membrane and inactive bacterial proteins, resulting in bacterial death [49]. On the other hand, released Ag^+^ penetrates the bacterial cell walls, results in structural changes, and damages the inner and outer bacterial structure [50].

**Figure 14:**
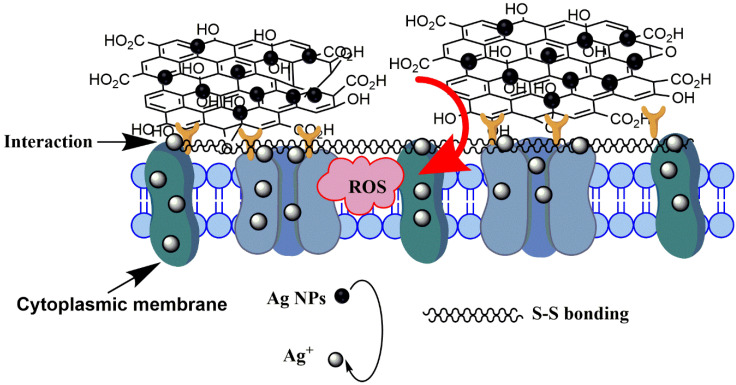
Proposed mechanism of interaction between *E. coli* and large GO–Ag NPs.

The third reason is the high ROS production induced by >1 µm GO–Ag NPs. ROS damage membrane-bound enzyme function, deactivate cellular enzymes and DNA, and inhibit the development of bacterial colonies. Moreover, the FTIR spectra in Figure 11 of both ~100 nm and >1 µm GO–Ag NPs exhibit intense peaks attributed to disulfide (S–S) bond vibrations, possibly because the nanometal may disturb the boundary of the defense barrier of the bacterial membrane [51]. The interaction between GO support and aromatic molecules occurs through π-π stacking. An interaction occurs between the polyaromatic domain and analytes on the GO surface, which works as a suitable platform for molecular trapping [52]. After treatment with GO, the bacterial membrane lost integrity as compared with the control *E. coli* (Figure 9). The GO functional groups, which bind with amino acids, have slightly changed. This phenomenon was indicated by a reduced intensity of C=O groups when combined with a $-{\mathrm{PO}}_{4}^{-3\;}$ group (secondary amino acid in *E. coli* membrane). Figure 14 summarizes the hypothesized mechanism of large GO–Ag NPs inactivating *E. coli*. In contrast, smaller GO–Ag NPs release fewer Ag^+^ ions, resulting in few Ag^+^ contact with the bacteria, low ROS generation and bactericidal effect. Therefore, the antibacterial activity of small GO–Ag NPs were proposed to be the disturbance of GO sheets and Ag^+^ ion release, whereas that of larger GO–Ag NPs was proposed due to a combination of wrapping by GO sheets, significant Ag^+^ ion release and ROS production. 

Table 2 summarizes the method, size category and mechanism of action from selected articles. Previous studies reported multiple mechanisms of action, including physical and chemical effects. Our antibacterial results are in agreement with those studies [14,15,18]. Large GO sheets exhibit more antibacterial effects than the small GO sheets in the solution phase. We proposed that, in addition to the trapping mechanism, the intensity of FTIR spectral peaks attributed to –COOH and $-{\mathrm{PO}}_{4}^{-3\;}$ reactive groups and disulfide bonding (S–S) was higher in the spectra of large GO than that in the spectra of small GO. More investigations on gram-positive bacteria using GO and GO–Ag NP composites is suggested.

## 4. Conclusions

In summary, probe sonication was used to fabricate GO and GO–Ag NPs with controllable sizes. Both quantitative and qualitative antibacterial activities were examined by time-dependent antibacterial activity and live/dead bacterial assays against *E. coli*. The results showed that >1 µm GO had a greater antibacterial effect than the ~100 nm GO. As seen in the TEM images, the *E. coli* were wrapped by large GO sheets, whereas the small GO caused a disturbance of the *E. coli* cells without totally isolating them from the nutrition medium. The larger GO–Ag NPs showed higher antibacterial activity than the smaller GO–Ag NPs. Mechanisms of the antibacterial behavior of large GO–Ag NPs were proposed based on chemical and physical properties. Firstly, large GO sheets physically wrap and inhibit the growth of *E. coli*. Secondly, Ag^+^ is released rapidly accumulating at the membrane surface and then penetrating into the cell interior. ROS production, which correlates with redox reactions, results in more cell death. In contrast, the ~100 nm GO–Ag NPs showed less bactericidal effects due to low ROS production and lower Ag^+^ release. This potential finding provides the size effect role, but also creates a foundation for a number of further biomaterial applications. This work also demonstrates valuable current insight and excitement to conquer new areas, such as waste water treatment, bacteria resistance or prevent biofilm formation.

## Figures and Tables

**Table 1:** Nanomaterial size and ζ-potential of graphene oxide (GO) with different sizes in aqueous solution (transmission electron microscopy (TEM) images were calculated for 50 pieces of GO sheets for each batch).

GO	~100 nm	100 nm~1 µm	~1 µm	>1 µm
**DLS hydrodynamic size (nm)**	140.8 ± 8.8	320.6 ± 71.6	1098.7 ± 381.7	4605.3 ± 338.8
**TEM results (nm)**	69.0 ± 7.8	161.4 ± 44.1	1005.2 ± 378.9	4340.1 ± 1189.1
**ζ-potential (mV)**	−35.1 ± 2.2	−36.7 ± 10.2	−33.1 ± 2.2	−36.7 ± 5.3

**Table 2:** A summary of GO’s various size-dependent antibacterial effects.

Lateral Size	Preparation	Characterization of Size	Bacteria/Cell Inactivation Results	Mechanism of Action (Finding)	Reference
0,.753, 0.127, 0.065, 0.035, 0.013, 0.01 µm^2^	Preparation for 0, 10, 30, 50, 120 and 240 min	- AFM	- Largest size GO showed 99% inhibition rate whereas around 56.2% for smallest size GO.	- Large GO can cover *E. coli*	[14]
0.65, 0.29, 0.1 and 0.01 µm^2^	Preparation for 0, 1, 10 and 120 min	- SEM	- Dispersion phase: larger >smaller size. - Coated membrane phase: smaller > larger size	- Dispersion phase: *E. coli* was inactive when entrapped by large GO - Membrane phase: Small GO caused loss of GSH than large GO.	[18]
GQD ( <15 nm), SGO (50–200 nm) and LGO (0.5–3 µm)	Commercial products	- AFM	- *E. coli*: LGO reduced viability by 30% > SGO (5.3%) > GQD (no effect) - *Murine cell*: LGO reduced viability > SGO > GQD	- Destruction by LGO and SGO but not by GQD.	[15]
SGO (30 nm), MGO (300 nm) and LGO (1 µm)	Synthesis using Hummer method from different size of graphites	- AFM and DLS	- SGO (78%) > LGO (76.4%) > MGO (69.1%) for bacterial strains (*Tox2).* - Toxicity effect on zebrafish was LGO > MGO > SGO	- ROS production by bacteria strain was LGO < MGO < SGO.	[17]
~100 nm, 100 nm to 1 µm, ~1 µm and >1 µm	Preparation for 0, 30, 60 and 120 min	- TEM and DLS results	- 17.8%, 30.1%, 41.8% and 47.9% for ~100 nm, 100 nm to 1 µm, ~1 µm and >1 µm GO - 27.6%, 80.6%, 83.2% and 88.6% for ~ 100 nm, 100 nm–1 µm, ~1 µm and >1 µm GO–Ag NPs	- LGO entrap E. coli. - LGO–Ag exhibited more Ag+ release than U-SGO–Ag - LGO induce more ROS	This study

Notes: GQD: graphene quantum dots; LGO: large GO; MGO: medium GO; SGO: small GO; GSH: glutathione; SEM: scanning electron microscope; AFM: atomic force microscopy.

## Supplementary Materials

The following are available online at https://www.mdpi.com/2079-4991/10/6/1207/s1, Figure S1: FTIR (a) and XRD (b) analysis of GO, Figure S2: FTIR (a) and XRD (b) analysis of GO–Ag NPs, Figure S3. Antibacterial activities of GO during 13 h observation (a) and GO–Ag NPs (b) during 4 h observation.
