# Supplementary material for: Size-Dependent Antibacterial Activity of Silver Nanoparticle-Loaded Graphene Oxide Nanosheets
Source: Nanomaterials (Basel). 2020 Jun 20;10(6):1207. doi: 10.3390/nano10061207 (PMC7353109; doi:10.3390/nano10061207)
Supplement: Supplementary file 1 [file nanomaterials-10-01207-s001.pdf]

Supporting Information

Size-Dependent Antibacterial Activity of Silver Nanoparticle-Loaded Graphene Oxide Nanosheets

Truong Thi Tuong Vi [1], Selvaraj Rajesh Kumar [1], Yu-Tzu Huang [2], Dave W. Chen [3], Yu-Kuo Liu [1] and Shingjiang Jessie Lue [1,3,4,*]

[1] Department of Chemical and Materials Engineering, Chang Gung University, Taoyuan City 33302, Taiwan; truongthituongvi005@gmail.com (T.T.T.V.); rajeshkumarnst@gmail.com (S.R.K.); ykliu@mail.cgu.edu.tw (Y.-K.L.)

[2] Department of Chemical Engineering, Chung Yuan Christian University, Taoyuan City 32023, Taiwan; yt_huang@cycu.edu.tw

[3] Department of Orthopedic Surgery, Chang Gung Memorial Hospital, Keelung City 20445, Taiwan; mr5181@cgmh.org.tw

[4] Department of Safety, Health and Environment Engineering, Ming-Chi University of Technology, New Taipei City 24301, Taiwan

* Correspondence: jessie@mail.cgu.edu.tw; Tel.:+866-3-211-8800 (ext.5489); Fax: +886-3-211-8700

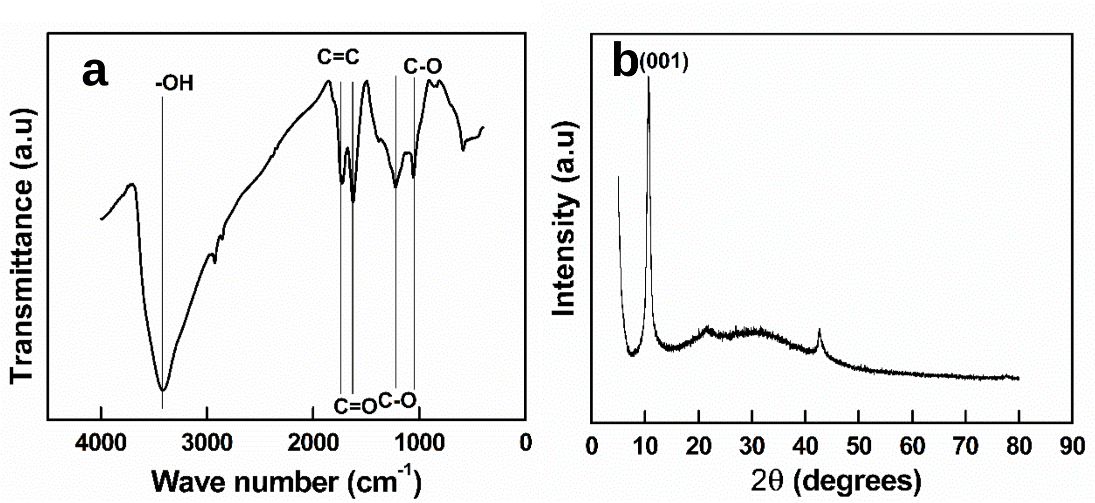

Figure S1. FTIR (a) and XRD (b) analysis of GO

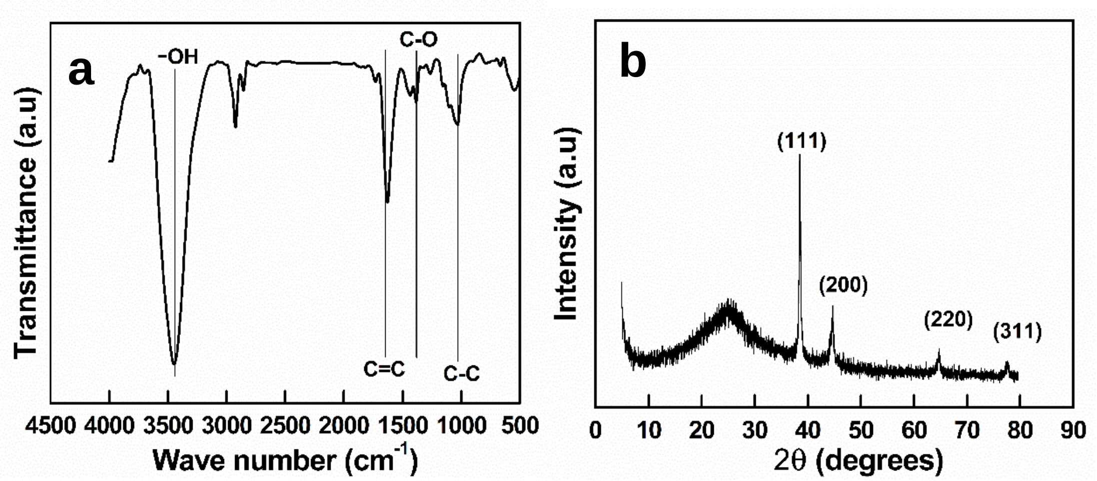

Figure S2. FTIR (a) and XRD (b) analysis of GO–Ag NPs

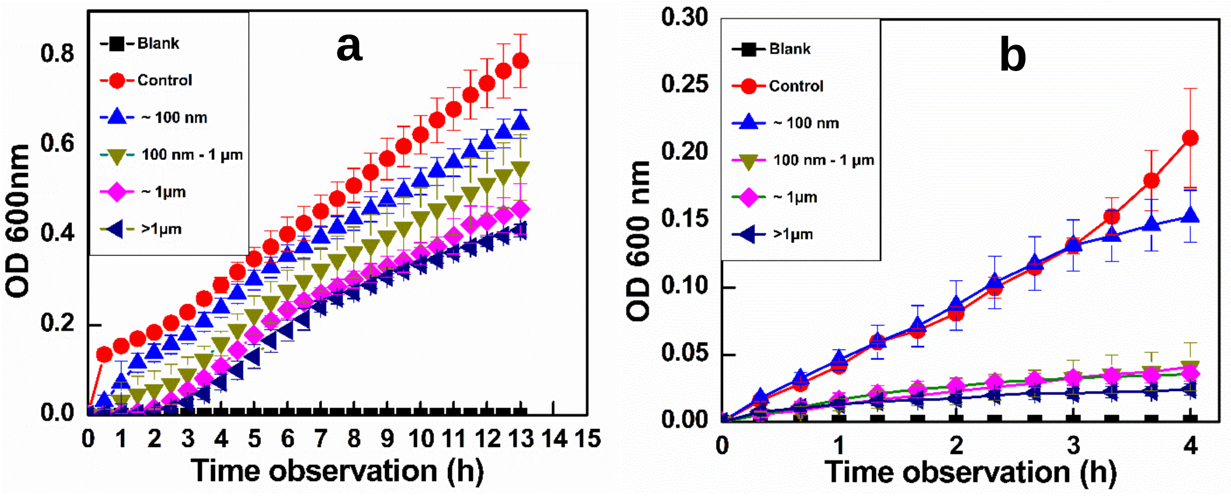

Figure S3. Antibacterial activities of GO during 13 h observation (a) and GO–Ag NPs during 4 h observation (b).
